# Vitamin E and N-Acetylcysteine as Antioxidant Adjuvant Therapy in Children with Acute Lymphoblastic Leukemia

**DOI:** 10.1155/2009/689639

**Published:** 2009-10-20

**Authors:** Youssef Al-Tonbary, Mohammad Al-Haggar, Rasha EL-Ashry, Sahar EL-Dakroory, Hanan Azzam, Ashraf Fouda

**Affiliations:** ^1^Hematology/Oncology Unit, Mansoura University Children's Hospital, Faculty of Medicine, Mansoura University, 35516 Mansoura, Egypt; ^2^Forensic Medicine and Clinical Toxicology Department, Faculty of Medicine, Mansoura University, 35516 Mansoura, Egypt; ^3^Clinical Pathology Department, Faculty of Medicine, Mansoura University, 35516 Mansoura, Egypt

## Abstract

Although cancer therapies have experienced great success nowadays, yet the associated toxic response and free radicals formation have resulted in significant number of treatment-induced deaths rather than disease-induced fatalities. Complications of chemotherapy have forced physicians to study antioxidant use as adjunctive treatment in cancer. This study aimed to evaluate the antioxidant role of vitamin E and N-acetyl cysteine (NAC) in overcoming treatment-induced toxicity in acute lymphoblastic leukaemia (ALL) during the intensive period of chemo-/radiotherapy, almost the first two months of treatment. Forty children newly diagnosed with ALL were enrolled in this study. Twenty children (group I) have taken vitamin E and NAC supplementations with chemotherapy and the other twenty children (group II) have not taken any adjuvant antioxidant therapy. They were evaluated clinically for the occurrence of complications and by the laboratory parameters (blood levels of glutathione peroxidase (Glu.PX) antioxidant enzyme, malondialdehyde (MDA), tumor necrosis factor-*α* (TNF-*α*), liver enzymes, and bone marrow picture). Results revealed reduced chemotherapy and radiotherapy toxicity as evidenced by decreasing level of MDA, increasing level of Glu.Px and decreased occurrence of toxic hepatitis, haematological complications, and need for blood and platelet transfusions in group I compared to group II. We can conclude that vitamin E and NAC have been shown to be effective as antioxidant adjuvant therapy in children with ALL to reduce chemo-/radiotherapy-related toxicities during the initial period of treatment.

## 1. Introduction

Cancer survivors are increasing nowadays; many of them are highly motivated to seek information about dietary supplement use and complementary nutritional therapies to improve their response to treatment [1]. Leukemia and lymphoma therapy, especially in children, is the success story of contemporary cancer treatment. There is widespread consensus that, with survival projections as good as they are, the emphasis is shifting towards improving tolerability and reducing side effects from treatment. Children undergoing treatment for acute lymphoblastic leukaemia (ALL) receive multiagent chemotherapy; many of them (cytosine arabinoside, doxorubicin, cyclophosphamide, and methotrexate) are associated with free radical production may affect the antioxidant status during therapy [2]. Also hepatic and hematological complications are very common due to multiagent chemotherapy especially during the initial intensive period of induction; usually these complications cause discontinuation of therapy, prolong the duration of stay in hospitals, and may affect the overall prognosis and outcome of the disease. There is a debate about the concurrent use of antioxidants with cytotoxic therapies [3]. It is true that much remains unknown concerning antioxidants, their mode of action, and possible interaction. Vitamin E (alpha tocopherol succinate) as antioxidant has generated some interest as an adjunctive cancer therapy due to its remarkable lack of toxicity in vivo [4]. If given with omega 3 fatty acids of fish oil, it may prolong survival in patients with generalized malignancy [5]. Another antioxidant is N-acetylcysteine (NAC) which is the acetylated form precursor of L-cysteine and reduced glutathione. It was used as a mucolytic agent in respiratory illness as well as an antidote for acetaminophen hepatotoxicity but recently used as complementary therapy of cancer [6, 7]. 

 This study aims to evaluate the role of vitamin E and N-acetylcysteine as adjuvant therapy in children with ALL during the induction and CNS intensification phases of chemotherapy, the initial two months of treatment, and their possible role in prevention and control of hepatic and hematological complications (bone marrow hypoplasia and febrile neutropenia) associated with the use of chemotherapeutic agents.

## 2. Patients and Methods

This study was conducted on a cohort of 40 children with ALL (18 males and 22 females) with their ages ranging between 2 and 15 years. They were enrolled consecutively from the recently diagnosed cases of ALL at Pediatric Hematology and Oncology Unit of Mansoura University Children's Hospital, Mansoura, Egypt, in the period between November 2006 and July 2007. Diagnosis of ALL was done according to standard methods including morphological, cytochemical, and immunological evaluation. Patients were treated by the modified BFM 76/79 protocol of therapy [8]**.** Cases with serious complications that may necessitate cessation of chemotherapy for undetermined duration were excluded from the selection. Patients were randomly allocated into one of two groups. First group (Group I) included 20 patients (10 males and 10 females) who were supplemented with vitamin E in a dose of 400 IU/day orally and N-acetylcysteine (NAC) in a dose of 600 mg/day orally [9] in addition to chemotherapy from day one of treatment till the end of CNS intensification (Phase II of chemotherapy, for a period of almost two months). CNS intensification phase includes a combination of chemotherapy (intrathecal methotrexate age-adjusted doses on days 4, 11, 18, and 25) and cranial irradiation (1800 cGy in 10 fractions of 180 cGy per day). The second group (Group II) included 20 patients (8 males and 12 females) who received chemotherapy and radiotherapy without any supplementation. Informed consent was obtained from the parents prior to giving chemotherapy and any supplementation. Blood samples were collected from every patient at day 28 of induction and at the end of CNS intensification for estimation of Malondialdehyde (MDA) thiobarbituric acid reactive substance, serum glutathione peroxidase (Glu.Px), tumor necrosis factor alpha (TNF-*α*), and liver function tests (SGOT, SGPT). Blood was collected into heparinised tubes which were protected from light and processed immediately after sampling. At the time of collecting the blood samples, patients were free of any potentially confounding or interfering conditions, such as infections, fever, derangements of glucose, and lipid metabolism. Patients were evaluated for occurrence of any complications, for example, haematological complications like bone marrow (BM) hypoplasia (BM cellularity less than 30 000 at the end of either induction or CNS intensification phases), febrile neutropenia, need for blood or platelet transfusions (hemoglobin level less than 8 gm/dL and platelet count less than 20 000/cmm, resp.), and toxic hepatitis (defined by elevation of liver enzymes, 2 to 3 folds after chemotherapy, with the exclusion of viral hepatitis and return to the normal range after suspending chemotherapy [10]).

### 2.1. Estimation of Serum Glutathione Peroxidase [11]

Glutathione peroxidase (Glu.Px) was assayed by kit purchased from Randox (Randox Laboratories Ltd., UK) (Cat. No. RS505). This method-based on the fact that is Glu.Px catalyses the oxidation of glutathione (GSH) by cumene hydroperoxide. In the presence of glutathione reductase (GR) and NADPH, the oxidized glutathione (GSSG) is immediately converted to the reduced form with a concomitant oxidation of NADPH to NADP. Then decrease in absorbance at 340 nm is measured calorimetrically.

### 2.2. Determination of Malondialdehyde (MDA) Thiobarbituric Acid Reactive Substances [12]

Serum proteins are precipitated by addition of trichloroacetic acid (TCA). Then, thiobarbituric acid (TAB) reacts with malondialdehyde (MDA) to form thiobarbituric acid reactive product, which is measured calorimetrically at 534 nm.

### 2.3. Determination of Human Tumor Necrosis Factor Alpha (TNF-*α*)

(TNF-*α*) is determined by ELISA technique (Biosource, Belgium) according to method of Beutler and Cerami [13].

### 2.4. Statistical Analysis

It was done by using SPSS for windows (Statistical Package for Social Science Version 10). Exploration of quantitative parameters revealed violation of normality in Glu.Px, MDA, and TNF-*α*, and liver enzymes for which nonparametric test (Mann Whitney U-Wilcoxon test) was applied with the use of median and interquartiles to express central tendency and dispersion. However frequency distribution of blood and platelet transfusions showed preserved normality; so *t*-test with mean and standard deviation was reasonable for the analysis of difference between the two groups of patients. Number and percentages of categorical parameters (frequency of hematological complications and toxic hepatitis) had been done with the application of Chi square test for analysis of the statistical significance. *P* is considered significant if ≤.05.

## 3. Results

Table 1 shows the median values and interquartiles of serum level of Glu.Px, MDA, and TNF-*α* in the two phases of therapy in both leukemia groups. There is a statistically significant increase in serum Glu.Px in group I (supplemented with vitamin E and NAC) compared to group II during phase II (*P* = .03). MDA is slightly decreased in group I after phase II of therapy but this decrease is statistically insignificant (*P* = .13). Serum level of TNF-*α* does not show any significant difference between both groups of patients after the two phases of therapy.

The occurrence of toxic hepatitis and the serum level of liver enzymes (SGOT and SGPT) are reported in Tables 2  and 3. The incidence of toxic hepatitis is significantly lower in group I of patients who were supplemented with vitamin E and NAC after both phases of therapy (*P* = .02 and *P* = .001, resp.). Similar results were found regarding the serum level of SGOT and SGPT (*P* = .007, .004, .022, and .003, resp.).

Hematological complications (bone marrow hypoplasia and febrile neutropenia) were statistically higher in group II of patients who received chemotherapy alone without any supplementation (*P* = .001) as shown in Table 4. Frequencies of blood and platelet transfusions are lower in group I compared to group II of patients after both phases of therapy (*P* = .001 for all differences) (Table 4).

## 4. Discussion

Many patients with cancer take antioxidant nutritional supplements during cancer treatment to alleviate treatment toxicities and to improve long-term outcomes, but little is known about the efficacy and safety of antioxidant use during cancer treatment [14]**. **Despite the considerable debate about the role of antioxidant status in cancer outcomes, very few studies have assessed changes in antioxidant status and oxidative stress. Among children undergoing treatment for cancer there was only one study, to our knowledge, that measured exposure and treatment outcome in pediatric oncology [15]. They found that children with ALL have altered antioxidant and micronutrient status at diagnosis and during treatment. Our results support this study; oxidative stress has been found to be increased during treatment and antioxidant status decreased as being measured by serum Glu.Px and MDA in the group of children with ALL who did not receive antioxidants. Antioxidant status was associated with treatment-related oxidative stress (febrile neutropenia and elevated liver enzymes). We observed a significant increase of serum Glu.Px in ALL patients receiving vitamin E and NAC after phase II of therapy in comparison to those who received chemotherapy alone without any supplementation. Although MDA decreased after phase II of therapy in group I of ALL patients, this decrease is not statistically significant; this could be explained, in part, by the relatively small sample size. Comparison of serum level of TNF-*α* did not show any difference between both groups of patients. These findings are consistent with the results of Portakal et al. [16], Ray et al. [17], and Mantovani et al. [18] who found an increase of oxidative stress during treatment in cancer patients as compared with control. Thus we may conclude that vitamin E and NAC supplementation had significantly decreased the level of free radicals resulting from oxidation as evidenced by increasing the level of GLu.Px and lowering level of MAD in ALL patients who took the supplementation.

The intensity of chemotherapy taken during induction phase and CNS intensification (vincristine, doxorubicin, cytosine arabinoside, cyclophosphamide, and 6-mercaptopurine) in addition to the prophylactic cranial irradiation which is proven to elicit 10 times more free radicals than chemotherapy alone leads to the increased incidence of toxic hepatitis as evidenced by increased serum levels of liver enzymes (SGOT and SGPT) and serum bilirubin. Occurrence of toxic hepatitis necessitates the interruption of treatment and prolongation of the period of hospital stay. Our study revealed significant decrease in the incidence of toxic hepatitis in group I compared to group II after induction chemotherapy (*P* = .02). Furthermore this difference increased more after CNS intensification phase of therapy (*P* < .001). Similar results had also been achieved as regard to the serum level of SGOT and SGPT in both groups of patients after the induction and CNS intensification phases of therapy (*P* = .007, .004, .022, and < .003, resp.). These results support the findings of Mantovani et al. [19, 20] who concluded the effective role of some antioxidants in reducing reactive oxygen species, proinflammatory cytokines levels, and increasing Glu.Px levels in cancer patients. The effective in vivo use of NAC as antioxidant to counteract chemotherapy and radiotherapy toxicity in cancer patients concomitantly with vitamin E confirmed the beneficial effect of its in vitro use in advanced stage cancer [21].

Hematological complications including bone marrow hypoplasia and febrile neutropenia as well as frequencies of blood and platelet transfusion were found to be significantly lower in group I compared to group II throughout the studied period (*P* < .001 for all differences).

Oxidative stress increases during treatment and antioxidant status decreases as measured by MDA and serum Glu.Px. With the combined use of NAC and vitamin E, some improvement in oxidative status had occurred; this could be considered as an encouraging result which paves the way for early combined use of these antioxidants as an adjuvant therapy for cancer chemotherapy. However further studies should be contemplated to assess the long term benefit of this adjuvant therapy during the maintenance chemotherapy, taking in consideration survival outcomes including disease-free and overall survival, and total chemo-/radiotherapy doses as basic parameters for evaluating the relative benefits and hazard ratios. This plan is of utmost importance, especially because of the feasible practical application of this adjuvant therapy being of low cost.

## Figures and Tables

**Table 1:** Serum glutathione peroxidase (Glu.Px), malondialdehyde (MDA), and tumor necrosis factor-*α* (TNF-*α*) in phases I and II of therapy for both leukemia groups.

Groups	Glu.Px (U/L)		MDA (nmol/mL)		TNF-*α* (pg/mL)	
	After phase I	After phase II	After phase I	After phase II	After phase I	After phase II
Group I						
Median	925	1142	3.52	2.79	41.17	40.34
I.Q	846–973	937–1409	2.79–4.14	2.1–3.4	28.5–72.3	13.4–82.3
Group II						
Median	949	936	3.57	3.32	39.29	36.25
I.Q.	810–1230	835–1012	2.84–4.23	2.75–4.42	22.1–59.9	18.9–71.8

*P*	.43	.03	.66	.13	.52	.85

I.Q.: interquartiles (25th–75th). Significance of Mann Whitney U-Wilcoxon Rank test if *P* < .05. Phase I: intensive induction chemotherapy. Phase II: CNS intensification phase.

**Table 2:** Toxic hepatitis in both leukemia groups during phases I and II of chemotherapy.

	Group I (*n* = 20)		Group II (*n* = 20)		*P*
	*No*	%	*No*	%	
After phase I	6	30.0	12	60.0	.02
After phase II	8	40.0	20	100.0	.001

Toxic hepatitis is diagnosed by elevation of liver enzymes 2-3 folds after chemotherapy with exclusion of viral hepatitis and normalization of these enzymes when chemotherapy was temporary suspended. Significance of Chi square test if *P* < .05.

**Table 3:** Liver enzymes levels of leukemia patients after phases I and II of chemotherapy.

	Group I (*n* = 20)	Group II (*n* = 20)	
Liver enzymes (U/L)	Median	Median	*P*
	I.Q.	I.Q.	
SGOT after phase I	82.39	144.2	.007
	56.2–112.4	124.3–182.4	
SGOT after phase II	197.4	380.4	.004
	96.8–216.5	246.6–490.5	
SGPT after phase I	137.58	224.1	.022
	122.1–156.2	189.4–264.2	
SGPT after phase I	169.5	772.5	.003
	145.8–199.4	508.4–821.6	

**Table 4:** Hematological complications (bone marrow hypoplasia and febrile neutropenia) as well as frequency of blood and platelet transfusions (during phases of chemotherapy) in both leukemia groups.

	Group I (*n* = 20)	Group II (*n* = 20)	*P*
Hematological			
complications N (%)	8 (40)	20 (100)	.001
Phase I (mean ± SD)			
*Blood transfusion*	1.44 ± 0.52	2.60 ± 0.69	.001
*Platelet transfusion*	1.20 ± 0.44	3.40 ± 1.50	.001
Phase II (mean ± SD)			
*Blood transfusion*	1.16 ± 0.40	2.20 ± 0.42	.001
*Platelet transfusion*	1.00 ± 0.00	1.60 ± 0.69	.001

Significance of Chi square test (for hematological complications) and *t*-test (for number of blood and platelet transfusions) if *P* < .05.
